# Patient self-management and pharmacist-led patient self-management in Hong Kong: A focus group study from different healthcare professionals' perspectives

**DOI:** 10.1186/1472-6963-11-121

**Published:** 2011-05-24

**Authors:** Fiona YY Wong, Frank WK Chan, Joyce HS You, Eliza LY Wong, EK Yeoh

**Affiliations:** 1 School of Public Health and Primary Care, The Chinese University of Hong Kong, Prince of Wales Hospital, Shatin, N.T. Hong Kong; 2 School of Pharmacy, The Chinese University of Hong Kong, Shatin, N.T. Hong Kong

**Keywords:** patient self-management, pharmacist-led patient self-management, chronic disease, health policy, Hong Kong

## Abstract

**Background:**

Patient self-management is a key approach to manage non-communicable diseases. A pharmacist-led approach in patient self-management means collaborative care between pharmacists and patients. However, the development of both patient self-management and role of pharmacists is limited in Hong Kong. The objectives of this study are to understand the perspectives of physicians, pharmacists, traditional Chinese medicine (TCM) practitioners, and dispensers on self-management of patients with chronic conditions, in addition to exploring the possibilities of developing pharmacist-led patient self-management in Hong Kong.

**Methods:**

Participants were invited through the University as well as professional networks. Fifty-one participants comprised of physicians, pharmacists, TCM practitioners and dispensers participated in homogenous focus group discussions. Perspectives in patient self-management and pharmacist-led patient self-management were discussed. The discussions were audio recorded, transcribed and analysed accordingly.

**Results:**

The majority of the participants were in support of patients with stable chronic diseases engaging in self-management. Medication compliance, monitoring of disease parameters and complications, lifestyle modification and identifying situations to seek help from health professionals were generally agreed to be covered in patient self-management. All pharmacists believed that they had extended roles in addition to drug management but the other three professionals believed that pharmacists were drug experts only and could only play an assisting role. Physicians, TCM practitioners, and dispensers were concerned that pharmacist-led patient self-management could be hindered, due to unfamiliarity with the pharmacy profession, the perception of insufficient training in disease management, and lack of trust of patients.

**Conclusions:**

An effective chronic disease management model should involve patients in stable condition to participate in self-management in order to prevent health deterioration and to save healthcare costs. The role of pharmacists should not be limited to drugs and should be extended in the primary healthcare system. Pharmacist-led patient self-management could be developed gradually with the support of government by enhancing pharmacists' responsibilities in health services and developing public-private partnership with community pharmacists. Developing facilitating measures to enhance the implementation of the pharmacist-led approach should also be considered, such as allowing pharmacists to access electronic health records, as well as deregulation of more prescription-only medicines to pharmacy-only medicines.

## Background

Chronic disease is a major cause of disability. Those with chronic conditions are significantly more likely to visit physicians, to be admitted as an inpatient, and to have longer stays in hospitals. All the above would lead to increases in health care expenditure [1,2]. Patient self-management or self-care is one of the key approaches to managing chronic disease [3]. It allows patients to take an active part in the management of their own condition [4]. Self-management programs usually cover techniques dealing with emotion, fatigue, pain, and isolation, in addition to encouraging appropriate exercise, nutrition and appropriate use of medications. It also includes communicating effectively with family, friends and health professionals, as well as the evaluation of new treatments [5-7]. Previous studies have shown that many of these self-management programs can improve patients' self-efficacy, self-rated health, cognitive symptom management, exercise behaviours and communication with physicians [4-6].

Patient self-management does not mean that patients would get less help from health professionals. A health professional would continue to have a partnership with patients to provide collaborative care to monitor their health conditions regularly, and to solve patients' problems. Studies have shown that pharmacists are the most accessible health care professional to many chronically ill patients [8] and therefore, they are desirable to lead patient self-management. In pharmacist-led patient self-management programs, pharmacists emphasize disease knowledge, medication counseling, self-management and monitoring of symptoms, lifestyle advice, and barriers to care [7,9-13]. They have been found clinically and statistically effective in improving disease parameters and quality of life of patients and reducing mortality.

The traditional role of pharmacists is to manufacture and supply medication [14]. More recently, however, the pharmacist's role has evolved into a patient-centered approach, and that has some doctors concerned about the threat to their medical professional status. A study found that physicians were most comfortable with pharmacists' responsibilities of catching prescription errors, providing patient education, suggesting non-prescription medications, and suggesting prescription medications to physicians [15]. Another study showed that doctors were happy to delegate tasks to pharmacists that they found difficult or mundane, such as monitoring drug adherence and repeat dispensing [16]. The doctors were more sceptical about pharmacists carrying out clinically orientated activities. Compared to the western countries, pharmacists' roles like prescribing, monitoring of disease parameters, health education, lifestyle modification, and smoking cessation counselling are limited in Hong Kong. The role of pharmacists in Hong Kong is primarily dispensing drugs.

In Hong Kong, patients with stable chronic conditions follow-up less frequently with their physicians, usually every four to six months. Patients may encounter health problems before their next follow-up appointment. Pharmacists may be suitable to lead patient self-management. The pharmacist-led approach is a collaborative care between pharmacists and patients, where they make healthcare decisions together [17]. Pharmacists could take some of the responsibilities from physicians like monitoring patients' disease parameter, assisting patients in solving health problems and developing self-management skills. However, the perspectives of physicians and other health care professionals on this issue should not be neglected.

In Hong Kong, in addition to western medicine, chronically ill patients may also seek advice from TCM practitioners and use Chinese medicines. In terms of patient self-management, self-medication and using western and Chinese over-the-counter medications are also common practice in our region. Physicians, pharmacists, TCM practitioners and TCM dispensers were targeted in this study because they had a crucial role in the western or Chinese medicine profession and are responsible for disease diagnosis and/or drug management. The aims of this study were to understand the perspectives of physicians, pharmacists, traditional Chinese medicine (TCM) practitioners and dispensers on self-management of patients with chronic conditions and the possibility in developing a pharmacist-led approach of patient self-management in Hong Kong.

## Methods

### Study Design

Participants were invited through the School of Public Health and Primary Care and the School of Pharmacy at The Chinese University of Hong Kong. Some of them were recruited through professional networks, especially those working in the private sector. Qualifications and working experience of all participants were ensured. Data collection was completed when the point of data saturation was reached. Nine homogeneous focus groups with 13 physicians (2 groups), 10 pharmacists (2 groups), 10 TCM practitioners (2 groups) and 18 TCM dispensers (3 groups) were conducted. The purpose and procedures of the focus group were explained. All identifying information was ensured to be kept confidential and informed consent was obtained from each of the participants. Before the discussion, a preamble introducing patient self-management with a case on diabetes mellitus was provided to facilitate the discussion. The moderators led the discussion based on a semi-structured discussion guide. The participants were encouraged to express their views freely. The duration of discussion was approximately 60-90 minutes and proceedings were audio-recorded and transcribed verbatim. A token of HK$200 was given to each participant as an appreciation.

### Subjects

A total of 13 physicians, 10 pharmacists, 10 TCM practitioners and 18 TCM dispensers participated in the study. They were academic or front-line workers, working in clinical and community setting. The demographic characteristics of the participants were shown in Table 1.

**Table 1:** Demographic characteristic of participants in focus groups

	Physicians	Pharmacists	TCM practitioners	TCM dispensers
Gender				
Male	8	4	7	9
Female	5	6	3	9
Age (yrs) (mean ± SD)	35.0 ± 7.72	27.6 ± 2.22	43.4 ± 20.33	33.8 ± 11.51
*Missing*	*1*			
Years of relevant experience (mean ± SD)	8.5 ± 6.91	4.6 ± 1.80	11.8 ± 15.30	10.4 ± 10.15
*Missing*	*5*		*2*	

### Instruments

A preamble introducing patient self-management and a case on diabetes mellitus were provided to the participants to facilitate their discussion. The moderators led the focus group discussions based on a semi-structured discussion guide which was derived from literature review [3,6,18-25] and further revised by experts to assure its content validity. A pilot of four homogeneous focus groups involving 11 medical and 12 pharmacy students was carried out. Appropriate adjustments were made before implementing the main focus groups. The final discussion guide consisted of open- ended questions emphasizing (1) Attitudes towards patient self-management; (2) Attitudes towards pharmacist-led self-management of patients with chronic conditions; (3) Extended roles of pharmacists on pharmacist-led patient self-management and (4) Collaboration between physicians, pharmacists and other health care professionals.

### Data Analysis

A five-stage data analysis in the framework approach was followed in the analysis: Familiarisation, Identifying a thematic framework, Indexing, Charting, and Mapping and interpretation [26]. The transcripts were analyzed independently by two investigators using the NVivo 7 software (QSR International Pty. Ltd. ^©^1999-2006). Broad themes were first identified. Emergent themes which occurred repeatedly across and within focus groups were noted as recurrent themes. Each theme was placed in a topic category based on its content. Large categories were further divided into sub-categories creating a tree-diagram. The two investigators discussed and examined the transcripts for connections among these themes until consensus was achieved. The master framework was applied to all the transcripts. Any changes in the framework and themes were discussed and agreed to on a repeated basis. Interpretations of the themes were illustrated by extracts from the transcripts.

The study protocol was approved by the Joint CUHK-NTEC Clinical Research Ethics Committee and was performed in accordance with the World Medical Association's Declaration of Helsinki.

## Results

### Attitudes towards patient self-management

Most of the physicians, pharmacists, TCM practitioners and dispensers agreed that patients had the responsibility to manage their own health. Some of them, especially the TCM practitioners and dispensers, stated that only patients whose health conditions were stable should be allowed to self- manage their own health (Table 2).

**Table 2:** Attitudes towards patient self-management and scope covered

Attitudes towards patient self-management
"...In the clinic, we cannot see the patients until a few months later when they come back for follow-up. What happened in these few months? We don't know. Patients can help themselves if they know how to manage their illnesses." (Physician)
"If the disease is in a stable condition, patients should be able to self manage their own health...But if the condition is unstable or they have complications, they may not be able to take care of themselves..." (TCM practitioner)
Scope covered in patient self-management
"For diabetic patients, for example, they need to know how to self monitor their blood sugar level, understand their disease and complications...need to know about diet control and other life style modification including smoking and alcohol." (Physician)
"...They need to know how to handle their medications, such as the functions, adverse effects and side effects,... adjust their living habits to minimize the risk of complications and be able to identify situations which need help from doctors..." (Pharmacist)
"...Especially those with diabetes, they need to have diet control otherwise medication doesn't help much. Besides, they need to have regular exercise and adequate sleep to maintain good health...." (TCM practitioner)
"It's a good idea to do self-management but many patients only know that they are sick but they don't understand the diseases they have... They don't know what they can do..." (TCM dispenser)
"...Patients' emotional status can influence their health. I think family support is important, especially for elderly patients..." (TCM dispenser)

### Scope covered in patient self-management

The four professions identified similar scope which should be covered in patient self-management. They believed that patients should have an understanding of their own disease, including symptoms and potential complications, and be able to perform disease parameter monitoring like blood pressure and blood glucose, drug management, side-effect monitoring and lifestyle modification if necessary. Most importantly, patients needed to know when they should seek help form health professionals. Support to caregivers was also identified as an important component in patient self-management. Many TCM practitioners and dispensers tended to focus more on quality of life, including sleeping quality and emotional support (Table 2).

### Attitudes towards pharmacist-led patient self-management and extended role of pharmacists

When the focus group participants were asked whether a pharmacist-led approach could be incorporated in patient self-management, the majority of physicians and TCM practitioners and dispensers believed that pharmacists were drug experts only and could only play an assisting role.

Nearly all the medical doctors were opposed to a greater role being played by community pharmacists. They believed that doctors were more competent and more experienced. Their perception was that pharmacists did not have sufficient knowledge to handle patients' clinical problems and that they were not as efficient as physicians in performing tasks other than handling drugs. Even if pharmacists were allowed to lead patient self-management, they still needed to develop trust with patients first. Similarly, the majority of TCM practitioners believed that medical doctors should play the leading role. Community pharmacists should be responsible for drug management only (Table 3).

**Table 3:** Attitudes towards pharmacist-led patient self-management and extended role of pharmacists

"Pharmacists can be part of the team but I don't think they can play a leading role. Physicians are still the leaders in disease management...Pharmacists have an important role in managing drug issues but they are not capable to give advice on other aspects." (Physician)
"I think both disease diagnosis and medications are important. Pharmacists are drug specialists but they are not as competent as physicians in disease diagnosis and treatment..." (TCM practitioner)
"...They are running a business. I doubt whether pharmacists would ask patients to go back to consult doctors. They need to sell their products."(TCM dispenser)
"Some patients don't know much about lifestyle modification...I can advise them what kinds of high cholesterol foods they should avoid... When they come over to buy blood pressure meters, I can teach them how to monitor their BP. All these services are free..." (Pharmacist)
"I agree with the pharmacist-led approach. Pharmacists can be the first point of contact and make appropriate referrals for patients... We can have other roles in addition to drug management but the problem is what kinds of support we can get." (Pharmacist)
"The government should shift the focus back to primary health care...We, pharmacists have a main role in primary health care and we can play an important role in disease prevention." (Pharmacist)

Almost all TCM dispensers stated that community pharmacists were drug experts only and they could not make clinical diagnosis. In a community pharmacy setting, pharmacists might have difficulties in counselling patients as patients' privacy and their information confidentiality had to be considered. Another concern is that pharmacies are profit-making businesses. TCM dispensers were worried that when patients had health concerns, community pharmacists would sell over-the-counter or health products for self treatment rather than referring patients back to physicians (Table 3).

In contrast to the other three professionals, all community pharmacists believed that they had extended roles and they were capable of leading patient self-management. They could be the first point of contact since pharmacists were accessible and their counselling services were free. Similar to physicians and TCM dispensers, most of the pharmacists also agreed that a good relationship and trust had to be developed with patients first, as many patients had the perception that pharmacists were only responsible for selling drugs. Nearly all agreed that support from the government is important, including funding and adjustment of regulations, to extend their roles and facilitate pharmacist-led patient self-management (Table 3).

### Collaboration between physicians, pharmacists and other health care professionals

Nearly all physicians were willing to collaborate with pharmacists, but only on medication issues. Physicians would still be the ones prescribing medications while pharmacists could suggest the most desirable medications and assist in monitoring patients' drug interaction and side-effects. Some facilitating factors like electronic patient record system, relevant training and supervision on pharmacies are necessary if pharmacists are to be involved in patient self-management (Table 4).

**Table 4:** Collaboration between physicians, pharmacists and other health care professionals

"Pharmacists can provide feedback to the patients' physicians and suggest which medications are more appropriate, rather than asking the patients to change the prescriptions directly. Usually there are some clinical reasons why the medication is prescribed." (Physician)
"Each patient should have a list or we need to have a system to record what kinds of drugs the patient are taking...Some patients consult numbers of doctors and take many medications..."(Physician)
"We can share tasks like education and lifestyle modifications with physicians. And if we notice that the patients have problems, we can refer them back to their doctors ... We can ask the TCM practitioners or dispensers what Chinese medicines and treatments the patients are using, but as there is still no concrete evidence to show the interactions between western and Chinese medicines, this would inhibit our collaboration ..." (Pharmacist)
"In medical and Chinese medicine trainings, students of both disciplines need to have some basic understanding about each other... I would give my opinions on Chinese medicine to patients, but if they have questions on western medicines and treatments, I would encourage patients to ask their physicians. As divergence of views between western and Chinese medicines exists, we better focus on our own area." (TCM dispenser)

The majority of pharmacists stated that it was necessary to collaborate with physicians, TCM practitioners, nurses, diabetic nurses, dieticians and social workers in handling chronic cases. A more comprehensive management plan could be developed if such a team could be formed. A centralized database is also needed to facilitate access to patients' information and communication. Some pharmacists also anticipated difficulties in working with TCM practitioners and dispensers because of the differences in western and Chinese medicines and treatments (Table 4).

Many TCM practitioners believed that collaboration with the western medicine profession was necessary. Many patients with chronic illness would consult TCM practitioners for long-term health maintenance. And if a patient's condition improved, a TCM practitioner might have to contact the physician and suggest lowering the patient's drug dosage. The majority of TCM practitioners and dispensers believed that it would be difficult to collaborate with western medical professionals because the medical concepts of western and Chinese varied. It was important to break the wall between the two professions before communication and cooperation could be facilitated. Physicians and pharmacists needed to understand more about the concept and theory behind Chinese medicine while TCM practitioners and dispensers also needed to learn how to read patients' medical records and understand the interactions between western and Chinese drugs (Table 4).

## Discussion

Generally, the four professions agreed that patients with chronic diseases should self-manage their illnesses, provided their health conditions were stable and they had sufficient knowledge of their diseases. Like many patient self-management programs conducted in other countries, they suggested that patients should obtain some basic disease information including symptoms, complications and treatments, followed by drugs and follow-up compliance and healthy lifestyle establishment [4,7,9-11].

Pharmacists believed they were capable of leading self-management of patients with chronic conditions, and could monitor patients' disease condition and parameters. They also believed they could provide health education and assist patients in lifestyle modification, as well as manage patients' drug issue. However, the other three professions believed that pharmacists could only play an assisting role, primarily in drug management. Pharmacists, therefore, could not lead patient self-management and the TCM practitioners suggested physicians take the lead. Similar to the general consensus of pharmacists in Hong Kong, the pharmacists that participated in the focus group believed they should play extended roles in health care [8]. They can be educators on self-care and advisors on implementing self-care. They can also assist patients in disease monitoring and lifestyle modification. Pharmacists are also specialists in following up patients' prescriptions, as well as provisions of drug counselling and formulating pharmaceutical care plans. For minor ailments like stomach ache, motion sickness or cold, pharmacists can also act as drug advisors. The physicians, TCM practitioners and dispensers in our study admitted that pharmacists could perform drug- related tasks better than physicians. However, they did not think delegating other tasks to pharmacists is appropriate. This finding was in agreement with other studies investigating opinions of physicians on the role of pharmacist [15,18,19,27]. They were worried that pharmacists did not have the relevant training and skills. They were not sure whether pharmacists were competent in roles other than handling drugs. This explains why many healthcare professionals have reservations about the pharmacist-led approach. The attitude of these three health professionals could be the result of a lack of familiarity with the pharmacy profession.

The four health professions also identified other barriers which inhibited the development of pharmacist-led patient self-management: the lack of a centralized patient record system for sharing and assessing patients' medical information, limited facilitating measures from the government, and the image of the pharmacy industry as a profit-making business. The pharmacist-led approach is not ready at the moment. It has to be developed gradually provided that the barriers that inhibit extending roles of pharmacists are minimized and more facilitating measures are developed.

The current health care system and structure of the service in Hong Kong is not conducive to pharmacists making their contributions. Therefore, it is not surprising that the focus group participants were concerned about their relationships with patients. Hong Kong has a dual system of registration for western and Chinese medicine. For western medicine, it is a public-private divide where primary care is mainly provided by the private sector and the majority of inpatient services are run by public sector. However, patients receiving health services from both the private and public sectors seldom have the opportunity to see community pharmacists. Those patients usually receive prescribed medications from private doctors directly or from government hospital/clinic pharmacies. Community pharmacists would only have the chance to provide consultation when patients visit pharmacies to buy drugs over the counter. It is difficult for them to follow-up with chronically ill patients regularly. Patients, therefore, are unsure about the role of pharmacists besides dispensing drugs and may not support pharmacist-led patient self-management. A study in Vietnam found similar results [28]. It found that pharmacists were considered medical doctors' assistants. Their role was to follow instructions while pharmacists in pharmacies earn money by selling pharmaceutical products to their customers but do not bother giving advice to the customers.

The pharmacists in the focus group claimed that the community pharmacy profession lacked support from the government. To promote the role of pharmacists, the government needs to reconsider enhancing the participation of pharmacists in health care service, especially in primary care. Traditionally, health care and treatment is provided by individual professional discipline. However, studies have supported the effectiveness of a multidisciplinary approach [29,30]. In Hong Kong, multidisciplinary patient care needs to be widely developed in the healthcare system. This would not only improve the quality of health services to patients but also ensure that our health professions are not underutilized. Both the health professionals and people in the community would have more exposure to other health care professionals rather than doctors and nurses alone. A public private partnership (PPP) can also be developed to encourage patients to consult community pharmacists for drug problems and to monitor drug compliance. By incorporating the PPP approach, the roles of community pharmacists can be promoted to the public, healthcare costs can be saved in the long term and drug incidents can also be minimized. In the long run, the government has to facilitate the reform of healthcare by considering the promotion of self-medication for minor ailments and the deregulation of more prescription- only medicines to pharmacy- only medicine [31].

To facilitate pharmacist-led patient self-management, the government needs to develop an electronic patient record system which can be accessed and updated by both medical doctors and community pharmacists to enable effective communication between the two professions and to improve the effectiveness of case management. To ensure quality of care of pharmacists, continuing pharmacy education has to be advocated. Pharmacists can be encouraged to obtain a certain knowledge level on Chinese medicine and patient self-management if credit transfer can be granted for previous learning in universities. Similarly, TCM practitioners and dispensers who are interested in taking courses related to western medicines or pharmacy may also be allowed a credit transfer. Equipment subsidized by the government for the screening and monitoring of patients' disease parameters in community pharmacies, as well as for patients to monitor their disease conditions at home are also needed.

As patient self-management involves collaboration of different health care workers, nurses, social workers and other allied health professionals should also be considered in future studies. Their views would also facilitate the development of patient self-management.

## Conclusions

Our study is the first one to explore the views of physicians, pharmacists, TCM practitioners and dispensers on patient self-management and the pharmacist-led approach. Self-management is beneficial to both patients and the healthcare system. As the Hong Kong population grows older, we will have more chronic illnesses to contend with, and these will inevitably cause increases in health spending. Patients with stable chronic conditions should participate in self-management in order to prevent the deterioration of their health and to save healthcare costs. With the consideration of insufficient medical and nursing staff and their heavy workloads, pharmacists who are well trained to manage minor ailments are considered to take the role in patient self-management. However, pharmacist-led patient self- management is not ready to be carried out. It has to be developed gradually with the support of government and the renovation of healthcare service delivery, including developing facilitating measures to extend pharmacists' responsibilities.

## Competing interests

The authors declare that they have no competing interests.

## Authors' contributions

FYW drafted the manuscripts, performed qualitative data analysis and interpreted the data and results. FWC was a moderator of the focus groups, performed qualitative data analysis and manuscript editing. JHY and ELW were moderators of the focus groups and performed qualitative data analysis. EKY performed manuscript editing. All authors were involved in the concept and designing the study and approved the final manuscript.

## Pre-publication history

The pre-publication history for this paper can be accessed here:

http://www.biomedcentral.com/1472-6963/11/121/prepub
